# Biodegradable Nanoparticles Mediated Co-delivery of Erlotinib (ELTN) and Fedratinib (FDTN) Toward the Treatment of ELTN-Resistant Non-small Cell Lung Cancer (NSCLC) via Suppression of the JAK2/STAT3 Signaling Pathway

**DOI:** 10.3389/fphar.2018.01214

**Published:** 2018-11-13

**Authors:** Donglai Chen, Fuquan Zhang, Jinhui Wang, Hua He, Shanzhou Duan, Rongying Zhu, Chang Chen, Lichen Yin, Yongbing Chen

**Affiliations:** ^1^Department of Thoracic Surgery, Shanghai Pulmonary Hospital, Tongji University School of Medicine, Shanghai, China; ^2^Department of Thoracic Surgery, The Second Affiliated Hospital of Soochow University, Suzhou, China; ^3^Institute of Functional Nano and Soft Materials, Jiangsu Key Laboratory for Carbon-Based Functional Materials and Devices, Collaborative Innovation Center of Suzhou Nano Science and Technology, Soochow University, Suzhou, China

**Keywords:** non-small cell lung cancer (NSCLC), erlotinib (ELTN), fedratinib (FDTN), JAK2 inhibitor, drug resistance, nano-delivery

## Abstract

**Background:** Erlotinib (ELTN)-based targeted therapy as first-line treatment for epidermal growth factor receptor (EGFR)-mutant lung cancers suffers from insufficient selectivity, side effects, and drug resistance, which poses critical challenges in the clinical setting. Acquired resistance of ELTN results in extremely poor prognoses of non-small cell lung cancer (NSCLC) patients, wherein activation of the JAK2/STAT3 signaling pathway has been proven to induce acquired ELTN resistance.

**Methods:** In this study, we developed a nanoparticle (NP) delivery system based on Food and Drug Administration (FDA)-approved poly(ethylene glycol) (PEG)-poly(lactic acid) (PLA) for the co-delivery of ELTN and fedratinib (FDTN, a small-molecular, highly selective JAK2 inhibitor). Both ELTN and FDTN could be encapsulated into the PEG-PLA NPs via optimization of the encapsulation method. The effect of NPs on NSCLC cells was evaluated by MTT assay. Western blotting was performed to study the molecular mechanisms of NPs inhibiting the downstream pathways of EGFR *in vitro*. The histological analysis and protein expression *in vivo* were assessed by hematoxylin/eosin (H&E) staining and immunohistochemistry, respectively.

**Results:** The drug cargoes exhibited great stability, and could be released more efficiently in the acidic tumorous condition. Mechanistic study showed that FDTN notably down-regulated the expression levels of proteins in the JAK2/STAT3 signaling pathway, including p-EGFR, p-JAK2, p-STAT3 and Survivin, therefore reversing the ELTN resistance. As a result, synergistic anti-cancer effect was achieved by PEG-PLA NPs encapsulating both ELTN and FDTN in ELTN-resistant NSCLC tumors both *in vitro* and *in vivo*, and lower systemic side effect was noted for the co-delivery NPs compared to free drugs.

**Conclusion:** This study provides a promising approach to overcome the ELTN resistance in the treatment of NSCLC, and the use of FDA-approved materials with clinically applied/investigated chemical drugs may facilitate the translation of the current delivery system.

## Introduction

Lung cancer is the most frequently diagnosed cancer and the leading cause of cancer death, with non-small cell lung cancer (NSCLC) being the most common type (Torre et al., 2015; Jamal-Hanjani et al., 2017). NSCLC is characterized by a number of gene point mutations, with approximately 70% of NSCLC patients experiencing somatic mutations in the exons of the epidermal growth factor receptor (EGFR) gene. NSCLC patients with the EGFR mutations respond well to the treatment with small-molecule EGFR tyrosine kinase inhibitors (EGFR-TKIs) (Lynch et al., 2004; Pao et al., 2004). Randomized trials have consistently demonstrated that EGFR-TKIs, such as erlotinib (ELTN), gefitinib, and afatinib feature better prognosis than conventional chemotherapy in distinct subgroup of advanced-stage NSCLC patients (Song et al., 2018). However, most patients, even those markedly responsive to initial treatment, develop resistance to EGFR-TKIs later on (Gazdar, 2009), posing a tremendous obstacle for the treatment of advanced-stage NSCLC.

STAT3 (signal transducer and activator of transcription (STAT) family protein-3) is primarily activated by tyrosine phosphorylation, which can be mediated by a number of tyrosine kinases including those of the Janus kinase (JAK) families (Looyenga et al., 2012). Constitutive STAT3 activation plays an important role in the resistance to small-molecule therapies that target oncogenes in NSCLC (Looyenga et al., 2012). A number of studies have reported that the activation of JAK2/STAT3 signaling pathway induces acquired ELTN resistance in lung cancer cells harboring EGFR mutation (Harada et al., 2012; Looyenga et al., 2012; Wu et al., 2013). Furthermore, JAK2 inhibition can sensitize resistant EGFR-mutant lung adenocarcinoma to tyrosine kinase inhibitors, which suggests the potential utilities of JAK inhibitors toward the treatment of EGFR-TKI-resistant NSCLC (Gao et al., 2016). Fedratinib (FDTN) is a small-molecular, highly selective JAK2 inhibitor, and has demonstrated therapeutic efficacy in patients with primary or secondary myelofibrosis (Pardanani et al., 2015). Therefore, we hypothesized that the combined use of ELTN and FDTN could enhance the cytotoxicity of ELTN and inhibit the growth of ELTN-resistant NSCLC cells via suppression of the JAK2/STAT3 signaling pathway.

In the clinical setting, ELTN is orally delivered, which has been claimed to result in side effects such as rash, diarrhea, gastrointestinal (GI) perforations, ocular lesions, and hematological disorders (Pardanani et al., 2015). Intravenous administration of ELTN at high dose is reported to be better tolerated than oral tablets (Kim et al., 2017). However, low aqueous solubility of ELTN greatly hurdles its application for systemic injection. Additionally, ELTN as a hydrophobic small molecule is easily eliminated from the body with low accumulation level to the tumor tissues. Similar to ELTN, FDTN also suffers from low solubility, and it causes adverse effects including nausea, vomiting, diarrhea, anemia, and thrombocytopenia (Marslin et al., 2009). Therefore, with the attempt to realize the cooperative anti-cancer effect of ELTN and FDTN against ELTN-resistant NSCLC, an effective delivery vehicle is highly demanded to enhance the drug solubility, promote tumor accumulation, and reduce side toxicity.

Nanoparticle (NP)-based delivery systems have been widely developed and studied for cancer therapy over the past decades, because of their desired capabilities to increase drug solubility, enhance drug stability, control drug release profiles, promote tumor accumulation, reduce toxicity, and overcome drug resistance (Pardanani et al., 2011; Seo et al., 2013; Kaur et al., 2015; Ramasamy et al., 2015; Zhou et al., 2015; Kulkarni et al., 2016; Kim et al., 2017). To date, most amphiphilic block co-polymers are based on the hydrophobic polyesters such as poly(lactic acid) (PLA) and hydrophilic poly(ethylene glycol) (PEG) (Ramasamy et al., 2014; Shen et al., 2017; Zhu et al., 2017). Among them, PEG-PLA (a U.S. Food and Drug Administration (FDA)-approved material) is the most widely used for drug delivery in cancer therapy. The PEG groups on the surface of the NPs impart long circulating and passive targeting properties (Tang et al., 2014; Dang et al., 2017; Wang et al., 2017), while the PLA segment displays desired biocompatibility as well as biodegradability (Yin et al., 2015). The paclitaxel formulation, Genexol-PM, based on PEG-PLA NPs was approved by South Korea in 2007 for the treatment of breast cancer, lung cancer, and ovarian cancer, and it is currently under clinical development in the United States (Lee et al., 2008; Luo et al., 2012). Several other nanomedicine formulations based on PEG-PLA NPs are also in clinical trials (He et al., 2016; Hofmann et al., 2017; Niu et al., 2017; Tao et al., 2017). Therefore, PEG-PLA would be an ideal material for the encapsulation of the lipophilic drugs such as ELTN and FDTN, which would endow better translational potentials.

In this study, PEG-PLA NPs were explored for their potentials to co-encapsulate ELTN and FDTN, and the capability as well as molecular mechanism of FDTN in reversing the ELTN resistance was unraveled. The *in vitro* anti-tumor efficacy of the co-delivery NPs in ELTN-resistant NSCLC cells (H1975 and H1650) was evaluated, and the *in vivo* efficacy was further validated in H1650 xenograft tumor-bearing mice.

## Experimental Section

### Materials, Cell Lines, and Animals

Poly(ethylene glycol) (PEG)-OH with molecular weight (MW) of 2 kDa was purchased from J&K (Beijing, China). D,L-lactide and Tin 2-ethylhexanoate were purchased from Energy Chemical (Shanghai, China). 1,2-Dipalmitoyl-sn-glycero-3-phosphoethanolamine (DGPE) and glacial acetic acid were purchased from Aladdin (Shanghai, China). ELTN and FDTN were purchased from Selleck Chemicals (Houston, TX, United States).

Non-small cell lung cancer cell lines, including H1650 (bearing a deletion in exon 19 of the EGFR gene, i.e., DE746-A750, EGFR 19delE746-A750) and H1975 cells (EGFR L858R/T790M, EGFR Exon21L858R+T790M+Exon20T790M) (ATCC, Rockville, MD, United States) were cultured in RPMI 1640 medium supplemented with 10% fetal bovine serum (FBS), 100 U/mL penicillin, and 100 U/mL streptomycin (Gibco BRL, Life Technologies, NY, United States).

Male athymic nude mice (4–6 weeks) were purchased from Shanghai Slaccas Experimental Animal Co., Ltd, and were housed in an SPF room. All animal study protocols were reviewed and approved by the Institutional Animal Care and Use Committee, Soochow University.

### Synthesis of PEG-PLA

Poly(ethylene glycol) (PEG)-OH (MW = 2 kDa) (100 mg) was dissolved in anhydrous toluene (9 mL). D,L-lactide (360 mg) and Tin 2-ethylhexanoate (20 mg) were added under water-free and oxygen-free conditions, and the solution was heated to 110°C for 24 h. Glacial acetic acid (0.3 mL) was then added into the solution to terminate the reaction. After cooling to room temperature, the reaction mixture was added dropwise into cold ether (40 mL). The solution was centrifuged at 5,000 rpm for 10 min and the precipitate was dried under vacuum. The white schistose solid product was obtained (80 mg), and its chemical structure was analyzed by ^1^H NMR (Supplementary Figure S1). MW distributions (polydispersity index, PDI = Mw/Mn) of the polymers were determined by gel permeation chromatography (GPC). The GPC analyses were performed on a Waters 1515 GPC instrument equipped with MZ-gel SDplus columns (500, 103, and 104 Ǻ) following a differential refractive-index detector (RI 2414). DMF with 0.05 mol L^-1^ LiBr was used as the eluant at a flow rate of 0.8 mL min^-1^ at 25°C.

### Preparation and Characterization of NPs

Poly(ethylene glycol) (PEG)-PLA, ELTN, and FDTN were separately dissolved in tetrahydrofuran (THF) at 20 mg/mL. A lipid, DGPE, was dissolved in chloroform (0.01 mg/mL). To an empty vial, lipid solution was added, and chloroform was evaporated by purging with nitrogen. To the same vial, the PEG-PLA solution (100 μL) was added at the polymer/lipid molar ratio of 95:5. ELTN and FDTN solution (20 μL) were then added. The mixture in THF were then added dropwise to a citrate buffer solution (pH = 4.0, 2 mL). The mixture was stirred for 1 h at room temperature, and THF was then evaporated by passing nitrogen through the mixture for 1 h. The formed NPs were then sonicated for 45 min using a bath sonicator, and dialysed against phosphate saline buffer (PBS, 10 mM, pH 7.4) for 4 h to remove un-encapsulated drugs. Drug encapsulation was determined by using high-performance liquid chromatography (HPLC) performed on a 1,200 Infinitely Series (Agilent Technologies, Santa Clara, CA, United States) with a UV detector, and an analytical C18 column (4.6 mm × 100 mm, 3.5 μm). The wavelengths for detecting ELTN and FDTN were 330 and 280 nm, respectively, using 0.1% TFA solution and acetonitrile (v/v = 7/3) as the mobile phase. The following equations were used to calculate the drug loading efficiency (DLE) and drug loading capacity (DLC).

$$\text{DLE}(\%)=\frac{\text{amount of drug encapsulated}}{\text{amount of drug added for encapsulation}}\times 100$$

$$\text{DLC }(\%)=\frac{\text{total weight of drug encapsulated in NPs}}{\text{total weight of NPs and drug encapsulated}}\times 100$$

The hydrodynamic diameters of NPs were measured using dynamic light scattering (DLS, Malvern Zetasizer Nano-ZS90). Measurements were conducted at a scattering angle of 90° using disposable polystyrene cuvette. An equilibration time of 120 s was maintained for all the measurements. The morphology of the NPs was observed by transmission electron microscopy (TEM, Tecnai G220).

The serum stability of NPs was determined by DLS. The NPs were diluted with DMEM containing 10% FBS for 10-fold. The particle size was monitored at different time points during the 2-h incubation period. To further evaluate the solution stability of NPs, both blank and drug-loaded NPs were incubated at RT for up to 7 days, wherein particle size was monitored every day.

### Drug Release From NPs

The release of ELTN and FDTN from PEG-PLA NPs was studied using a dialysis method in PBS (10 mM, pH 7.4 or 5.0). Briefly, drug-loaded NPs were put into the dialysis bag (MWCO = 1 kDa), and were dialyzed against PBS (10 mL) at 37°C and 150 rpm. At determined time intervals, 1 mL of the release medium was harvested and replenished with an equal volume of fresh medium. The amount of ELTN and FDTN in the harvested medium was determined by HPLC, and the cumulative drug release was calculated.

### *In vitro* Anti-cancer Efficacy

Exponentially growing NSCLC cells were seeded into 96-well culture plates at 1 × 10^4^ cells/well. After 24 h-culture cells were treated with PEG-PLA NPs encapsulating ELTN (ELTN@PEG-PLA), FDTN (FDTN@PEG-PLA), and ELTN+FDTN (ELTN+FDTN@PEG-PLA) at various drug concentrations for another 48 h. Cells treated with ELTN+FDTN (free drugs) were incorporated as control. Cells were also treated with drug-free NPs to assess the cytotoxicity of NPs materials. Cell viability was determined using the 3-(4,5-dimethylthiazol-2yl)-2,5-diphenyltetrazolium bromide (MTT) assay, and results were represented as percentage viability of control cells treated with PBS using the same method as described above.

### Western Blot Analysis

Cancer cells treated with NPs encapsulating ELTN, FDTN, or a combination therefore were lysed with the lysis buffer [50 mM Tris-HCl (pH 7.5), 250 mM NaCl, 0.1% NP40, and 5 mM EGTA containing 50 mM sodium fluoride, 60 mM β-glycerol-phosphate, 0.5 mM sodium vanadate, 0.1 mM phenylmethylsulfonyl fluoride, 10 μg/mL aprotinin, and 10 μg/mL leupeptin]. Protein concentration was measured using a BCA Protein Assay Kit (Thermo Scientific, Rockford, IL, United States). SDS-polyacrylamide gel electrophoresis (SDS-PAGE) was used to separate proteins. The SDS-polyacrylamide gels were transferred onto PVDF membranes (Millipore, Bedford, MA, United States). The membranes were incubated with specific primary antibodies against JAK2, phospho-JAK2 (p-JAK2), STAT3, p-STAT3, Survivin (Cell Signaling Technology, San Diego, CA, United States), and subsequently with HRP-conjugated secondary antibodies conjugated to Alexa Fluor 680, or IRdye 800 (Rockland Immunochemicals, Inc. Gilbertsville, PA, United States). The intensities of the protein bands were scanned using the Odyssey Infrared Imaging System (Li-Cor Biosciences, Lincoln, NE, United States).

### Anti-cancer Efficacy *in vivo*

H1650 cells (1 × 10^6^) were injected subcutaneously (s.c.) into the right flank of athymic nude mice. When tumor size reached approximately 100 mm^3^, mice were divided into five groups (eight mice per group), which respectively received intravenous (i.v.) injection of PBS, ELTN@PEG-PLA (6 mg/kg), FDTN@PEG-PLA (3 mg/kg), ELTN+FDTN@PEG-PLA (6 mg ELTN/kg and 3 mg FDTN/kg), and ELTN+FDTN (free drugs, 6 mg ELTN/kg, 3 mg FDTN/kg, dissolved in DMSO) on days 1, 3, 5, 7, 9, 11, and 13. Tumor sizes were measured by a caliper every week. Tumor volume (*V*) was calculated according to the following equation: *V*(mm^3^) = *a*^2^ ×*b* × 1/2 (*a*: length, *b*: width). Animal survival was also monitored within the observation period of 60 days.

### Histological Analysis

Three days post the last injection in the efficacy study described above, animals were euthanized, and liver and kidney tissues in each group were collected, fixed in 10% formalin, embedded in paraffin, and sectioned into 5-μm slices. The tissue sections were stained with hematoxylin/eosin (H&E) and observed under an optical microscope.

### Immunohistochemistry

Three days post the last injection in the efficacy study described above, animals were euthanized, and tumor tissues were harvested, fixed in 10% formalin, deparaffinized, rehydrated, and boiled in 0.01 M sodium citrate for antigen retrieval. The endogenous peroxidase activity was quenched. The sections were incubated with anti-p-EGFR (1:250, abcam) or anti-p-STAT3 (1:250, abcam) overnight at 4°C. Tumor sections were then incubated with biotinylated secondary antibodies (Dako, Glostrup, Denmark). Staining was visualized using diaminobenzidine (DAB). Representative photos were taken with a Nikon Eclipse E800 microscope equipped with a Nikon DXM1200 digital camera (Nikon instruments, Melville, NY, United States).

### Statistical Analysis

Statistical difference between two groups was evaluated with the Student’s *t*-test, and was assessed to be significant at ^∗^*p* < 0.05 and very significant at ^∗∗^*p* < 0.01 and ^∗∗∗^*p* < 0.001. The Kaplan–Meier method was used to analyze the survival time of tumor-bearing mice.

## Results and Discussion

### Preparation and Characterization of Drug-Loaded NPs

Poly(ethylene glycol) (PEG)-PLA was successfully synthesized via PEG-OH-initiated ring opening polymerization of D,L-lactide at the M/I ratio of 50, and its chemical structure was confirmed by ^1^H NMR (Supplementary Figure S1). By ^1^H NMR analysis, the MW of the PEG-PLA was calculated to be 7.9 kDa (polymerization degree of 78) with a PDI of 1.3.

**SCHEME 1:**
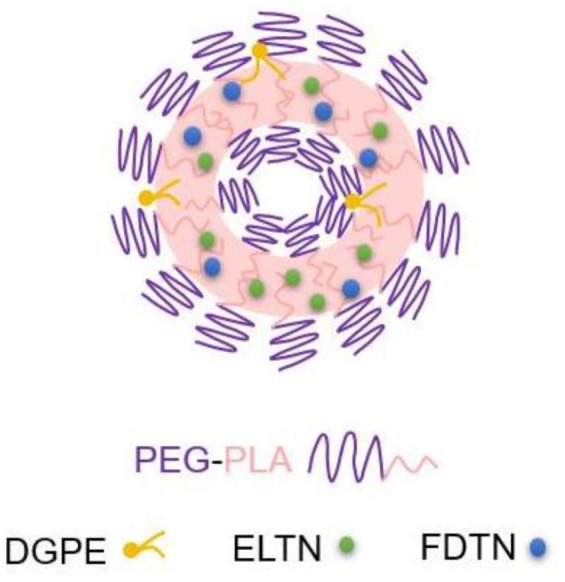
Structure of ELTN+FDTN@PEG-PLA NPs.

**FIGURE 1:**
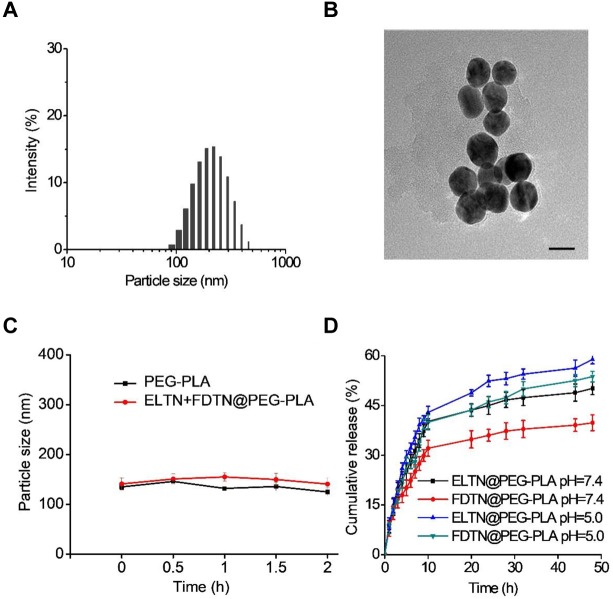
**(A)** Size distribution of ELTN/FDTN co-loaded NPs. **(B)** TEM image of ELTN/FDTN co-loaded NPs (scale bar = 200 nm). **(C)** Size alteration of NPs after incubation with 10% serum for different time (*n* = 3). **(D)** Cumulative release of ELTN and FDTN from NPs at pH 7.4 and 5.0 (*n* = 3).

Erlotinib and FDTN were encapsulated into PEG-PLA NPs via the solvent exchange method (Scheme 1; Kim and Lee, 2010). The introduction of the DGPE lipid could improve the stability of NPs and thus allowed more ELTN to be encapsulated (Table 1; Kulkarni et al., 2016). Furthermore, different aqueous phases were applied during the preparation of PEG-PLA NPs, and it was observed that using citric acid (pH 4.0) as the aqueous phases led to higher DLC than using PBS (pH 7.4) or HEPES (pH 7.4). As such, highest DLC of ELTN was achieved at 11.8% when citric acid and DGPE were used to prepare ELTN-loaded NPs. Using this formulation, ELTN and FDTN could be co-encapsulated with the DLC of 12.2 ± 2.1 and 4.2 ± 1.3%, respectively. Particle size of prepared NPs was ∼120 nm, as determined by DLS and TEM (Figures 1A,B). After incubation with 10% serum, particle size of both non-loaded and drug-loaded NPs maintained relatively constant within 2 h (Figure 1C), indicating desired serum stability of NPs that could potentially benefit their utilization for systemic administration. The NPs could also remain stable after incubation at RT for 7 days (Supplementary Figure S2), indicating their stability during storage.

**Table 1:** The DLC of ELTN in PEG-PLA NPs with different preparation conditions.

Entry	Buffer	Lipid (DGPE)	DLC (%)
1	Citric acid buffer (pH 4.0)	+	11.8
2	PBS (pH 7.4)	+	0.3
3	HEPES (pH 7.4)	+	0.1
4	Citric acid buffer (pH 4.0)	-	5.6

### *In vitro* Drug Release

*In vitro* drug release of ELTN and FDTN was evaluated at both pH 7.4 (the neutral peripheral blood and extracellular matrix) and pH 5.0 (the endolysosomal acidic microenvironment). As shown in Figure 1D, the cumulative release of FDTN was relatively low at pH 7.4 with an accumulative release amount of ∼35% within 48 h, while nearly 50% of the encapsulated FDTN was released at pH 5.0. Such pH-dependent release profile was also observed for ELTN. It may be caused by the protonation of the amine groups in ELTN and FDTN (Mandal et al., 2016), which enhanced the hydrophilicity of the drug molecules to facilitate their release from the hydrophobic domains of the NPs. Therefore, it is believed that the co-delivery NPs could steadily and efficiently release FDTN and ELTN, especially in the acidic microenvironment in the endolysosomes in tumor cells.

### *In vitro* Anti-cancer Efficiency Against ELTN-Resistant NSCLC Cells

**FIGURE 2:**
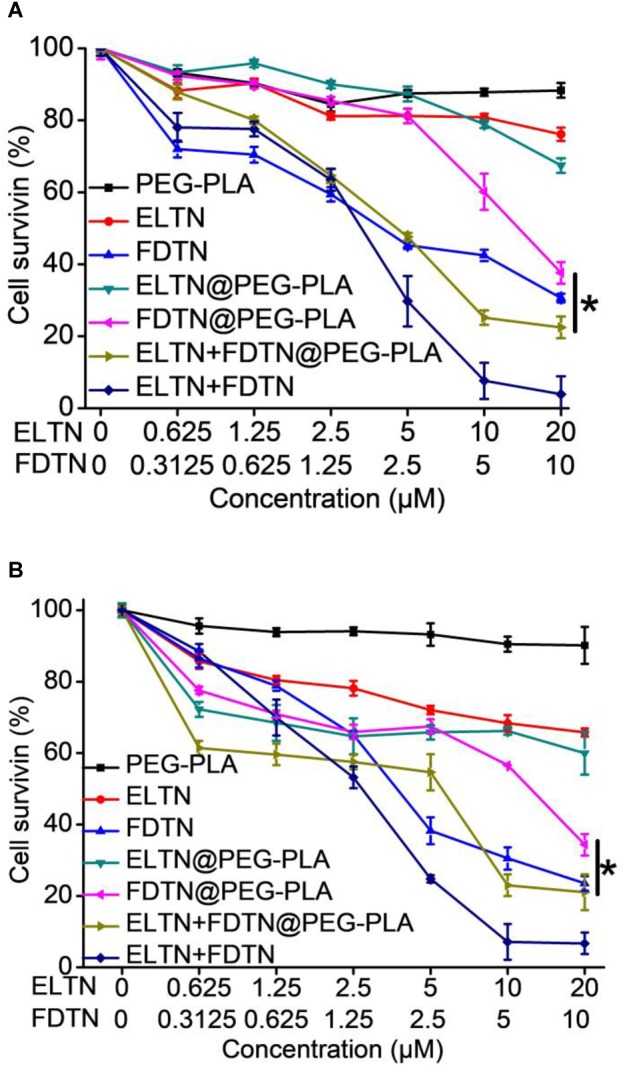
*In vitro* anti-cancer efficacy of PEG-PLA, ELTN, FDTN, ELTN+FDTN (free drug), ELTN@PEG-PLA, FDTN@PEG-PLA, and ELTN+FDTN@PEG-PLA in H1975 **(A)** and H1650 **(B)** cells at various drug concentrations following 48-h incubation (*n* = 3).

Human lung adenocarcinoma cell lines H1975 and H1650 that are resistant to ELTN were selected to assess the anti-cancer efficacy of drug-loaded NPs *in vitro* following 48-h incubation using the MTT assay. As shown in Figure 2 and Supplementary Table S1, the *in vitro* anti-cancer efficacy of drug-loaded NPs following 48-h treatment was evaluated and compared to free drugs using the MTT assay. Notably, co-delivery NPs showed superior cytotoxicity toward both ELTN-resistant cell lines than NPs individually encapsulating ELTN or FDTN, indicating that FDTN enhanced the cytotoxicity of ELTN toward ELTN-resistant lung cancer cells. The combination index (CI) between ELTN and FDTN were calculated to be lower than 1 (0.46 and 0.61 in H1975 and H1650 cells, respectively), indicating synergistic effect between these two drugs (He et al., 2018). Although the cytotoxicity of free drugs (ELTN+FDTN) was slightly greater than the co-delivery NPs, it could be attributed to the incomplete *in vitro* release of ELTN and FDTN from NPs in the absence of intratumoral acidic microenvironment. In comparison, drug-free NPs exhibited unremarkable cytotoxicity at high concentration up to 20 μM ELTN equivalent, which was reflective of the desired safety profiles of the NPs due to the use of biocompatible and biodegradable polymeric materials (Jiao et al., 2018; Lv et al., 2018; Ou et al., 2018).

### Regulation of JAK2/STAT3 Signaling Pathways

**FIGURE 3:**
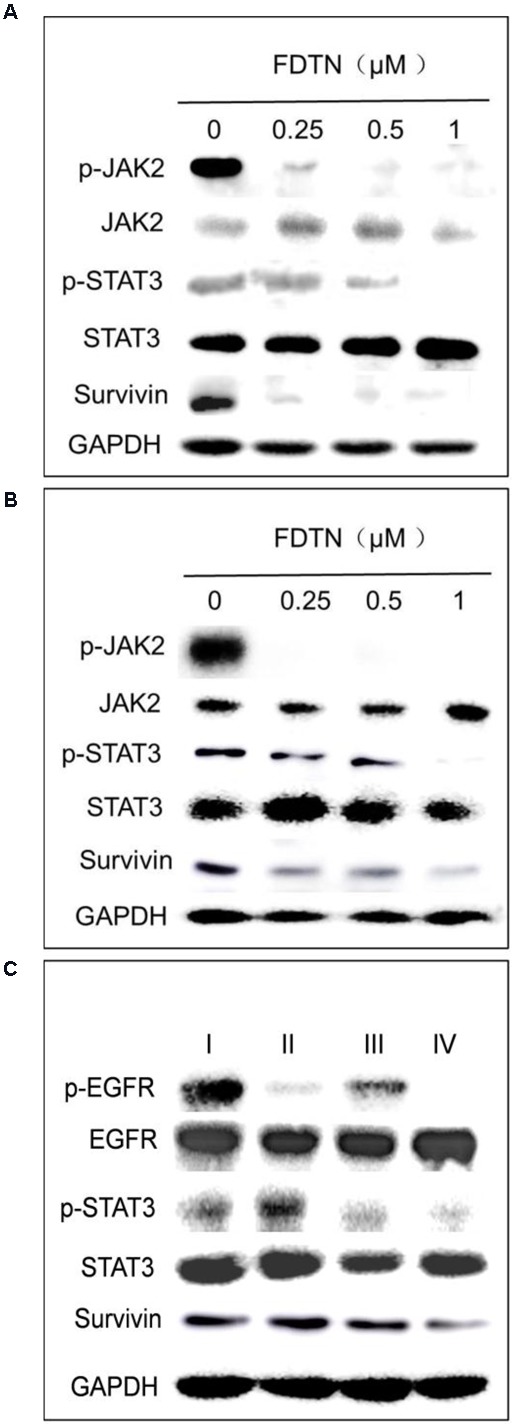
FDTN-loaded NPs regulate the expression of JAK2/STAT3 signaling pathway in H1975 **(A)** and H1650 **(B)** cell lines at different concentrations *in vitro*. **(C)** ELTN/FDTN co-loaded NPs downregulate p-EGFR and apoptosis-related proteins in H1650 cells *in vitro*. I: PEG-PLA; II: ELTN@PEG-PLA; III: FDTN@PEG-PLA; IV: ELTN+FDTN@PEG-PLA.

In order to investigate the effect of co-delivery NPs on the protein expression levels in cancer cells, Western blot was applied to determine the relationships between FDTN concentration and the expression of proteins in the JAK2/STAT3 signaling pathway in H1975 and H1650 cell lines. As shown in Figures 3A,B, the expression of p-JAK2, p-STAT3 and Survivin was down-regulated with increasing concentration of FDTN in both cell lines. In addition, the expression of p-EGFR, p-STAT3 and Survivin was down-regulated more significantly in lung cancer cells treated with co-delivery NPs than any other formulations (Figure 3C). These results collectively substantiated the potent efficacy of the co-delivery NPs in the down-regulation of JAK2/STAT3 signaling pathway.

### *In vivo* Anti-tumor Efficacy in ELTN-Resistant NSCLC

**FIGURE 4:**
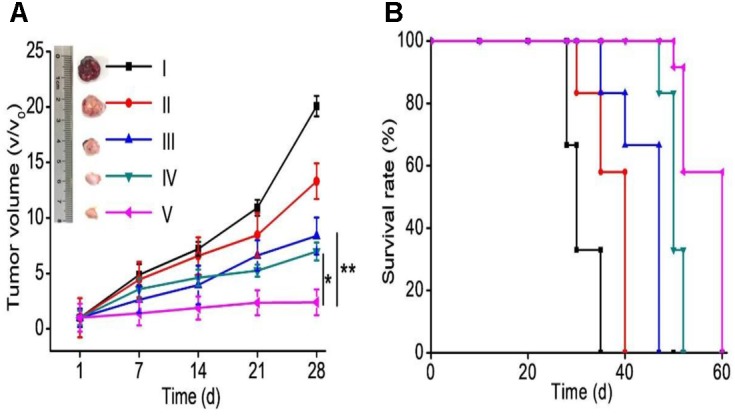
Tumor progression **(A)** and animal survival **(B)** following i.v. injections of PBS (I), ELTN@PEG-PLA (II), FDTN@PEG-PLA (III), ELTN+FDTN (free drug, IV), and ELTN+FDTN@PEG-PLA (V) in H1650 xenograft tumor-bearing mice at 6 mg ELTN/kg and 3 mg FDTN/kg on days 1, 3, 5, 7, 9, 11, and 13 (*n* = 8).

To further demonstrate the therapeutic efficacy against ELTN-resistant lung cancer cells *in vivo*, H1650 xenograft tumor models were established via subcutaneous injection. Mice were divided into five groups after the average tumor volume reached 80–100 mm^3^, and they subsequently received systematic treatment of different formulations. More significant tumor inhibition was found for the co-delivery NPs than free drugs or NPs individually encapsulating ELTN or FDTN (Figure 4A), although free drugs exhibited stronger cytotoxicity *in vitro* than co-delivery NPs. Tumors in mice treated with ELTN+FDTN@PEG-PLA almost stopped growing during the observation period of 28 days. In accordance, animal survival was significantly prolonged within the observation period of 60 days, wherein ELTN+FDTN@PEG-PLA again outperformed ELTN@PEG-PLA, FDTN@PEG-PLA, or ELTN+FDTN (Figure 4B). These results again demonstrated the effect of NPs delivery in potentiating the anti-cancer effect of chemodrugs, and FDTN enhanced the therapeutic effect of ELTN in ELTN-resistant NSCLC tumors.

### Histological Analysis

**FIGURE 5:**
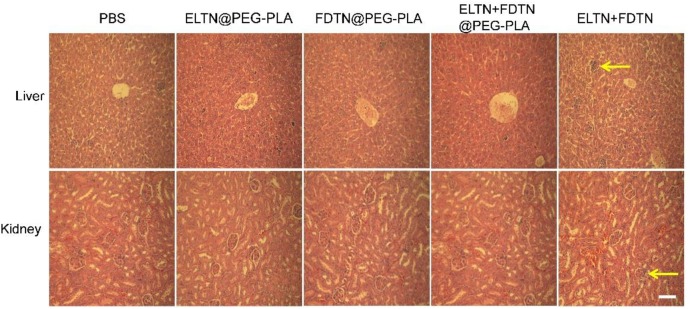
HE-stained liver and kidney sections following treatment with different formulations. Tissues were harvested on day 3 post the last injection. Bar represents 100 μm. Arrows indicate the region of necrosis of the liver and glomerular fibrosis.

The major organs of mice were sectioned and stained with H&E to assess the tissue damage. As shown in Figure 5, the damage and hyperemia of hepatic lobule in mice treated with co-delivery NPs was less serious than mice treated with the combination of free drugs. Additionally, mouse kidney after treatment with co-delivery NPs showed no remarkable structural abnormality of glomeruli compared to the appreciable glomeruli damage following treatment with free drug combination. These results indicated that by encapsulation of the drugs in NPs, the systemic toxicity could be greatly reduced.

**FIGURE 6:**
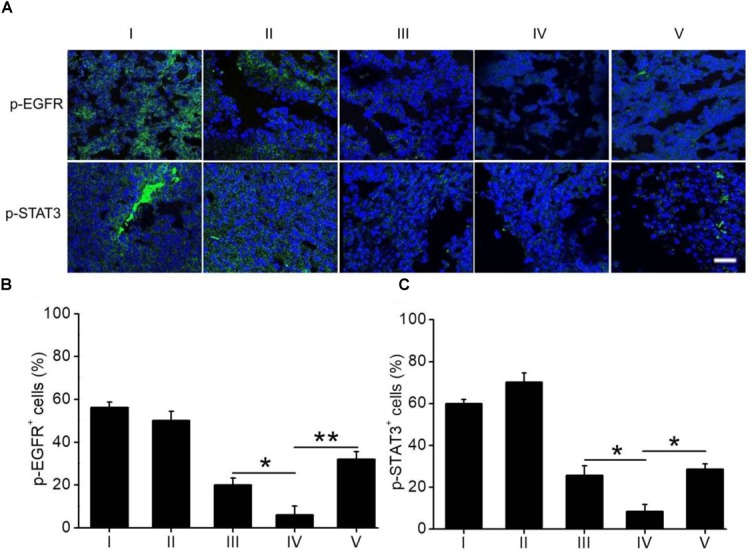
**(A)** Immunostaining of p-EGFR and p-STAT3 in tumor sections harvested on day 3 post the last injection of different formulations. Bar represents 100 μm. Quantification of the expression levels of p-EGFR **(B)** and p-STAT3 **(C)** from the immuno-stained images (*n* = 20). I: PBS; II: ELTN@PEG-PLA; III: FDTN@PEG-PLA; IV: ELTN+FDTN@PEG-PLA; V: ELTN+FDTN.

The expression of p-EGFR and p-STAT3 in H1650 tumors following drug treatment was monitored by immunostaining. As shown in Figure 6, the expression levels of both p-EGFR and p-STAT3 in mouse tumors treated with ELTN+FDTN@PEG-PLA were obviously lower than those in tumors treated with ELTN@PEG-PLA or FDTN@PEG-PLA alone, suggesting the synergistic anti-cancer effect between ELTN and FDTN via reversal of ELTN resistance in H1650 cancer cells. Treatment of ELTN+FDTN@PEG-PLA led to decreased expression level of p-EGFR and p-STAT3 than free drugs (ELTN+FDTN), which might be attributed to the long circulation and tumor targeting effect enabled by the NPs (Song et al., 2017; Yang et al., 2017; Jiao et al., 2018).

## Conclusion

We have successfully developed a co-delivery NPs system based on FDA-approved PEG-PLA for overcoming the ELTN-resistance toward targeted cancer therapy. We demonstrated that the NPs could encapsulate both hydrophobic ELTN and FDTN to enhance their delivery efficiency and therapeutic efficacy against ELTN-resistant NSCLC. FDTN contributed to the downregulation of the JAK2/STAT3 signaling pathway, and thus reversed the ELTN resistance to enable enhanced anti-tumor efficacy both *in vitro* and *in vivo*. Additionally, drug-loaded NPs also resulted in less toxicity than free drugs, suggesting the potential of this formulation for the treatment of ELTN-resistant NSCLC.

## Author Contributions

DC, FZ, HH, YC, and LY designed the study. DC, FZ, JW, SD, and RZ performed the experiments and analyzed the data. DC, FZ, JW, HH, SD, RZ, CC, YC, and LY wrote the manuscript.

## Conflict of Interest Statement

The authors declare that the research was conducted in the absence of any commercial or financial relationships that could be construed as a potential conflict of interest.
