# Supplementary material for: Biodegradable Nanoparticles Mediated Co-delivery of Erlotinib (ELTN) and Fedratinib (FDTN) Toward the Treatment of ELTN-Resistant Non-small Cell Lung Cancer (NSCLC) via Suppression of the JAK2/STAT3 Signaling Pathway
Source: Front Pharmacol. 2018 Nov 13;9:1214. doi: 10.3389/fphar.2018.01214 (PMC6242943; doi:10.3389/fphar.2018.01214)
Supplement: Supplementary file 1 [file Data_Sheet_1.docx]

Supporting Information

**Biodegradable Nanoparticles Mediated Co-delivery of Erlotinib (ELTN) and Fedratinib (FDTN) toward the Treatment of ELTN-Resistant Non-Small Cell Lung Cancer (NSCLC) via Suppression of the JAK2/STAT3 Signaling Pathway**

Donglai Chen^1,#^, Fuquan Zhang^2,#^, Jinhui Wang^3,#^, Hua He^3^, Shanzhou Duan^2^, Rongying Zhu^2^, Chang Chen^1,*^, Lichen Yin^3,*^, Yongbing Chen^2,*^

^1^ Department of Thoracic Surgery, Shanghai Pulmonary Hospital, Tongji University School of Medicine, Shanghai 200433, China

^2^ Department of Thoracic Surgery, the Second Affiliated Hospital of Soochow University, Suzhou 215004, China

^3^ Jiangsu Key Laboratory for Carbon-Based Functional Materials and Devices, Institute of Functional Nano and Soft Materials (FUNSOM), Collaborative Innovation Center of Suzhou Nano Science & Technology, Soochow University, Suzhou 215123, China

^#^ These authors contributed equally.

Address correspondence to:

Yongbing Chen ([chentongt@sina.com](mailto:chentongt@sina.com)); Lichen Yin ([lcyin@suda.edu.cn](mailto:lcyin@suda.edu.cn)); Chang Chen (chenthoracic@163.com)


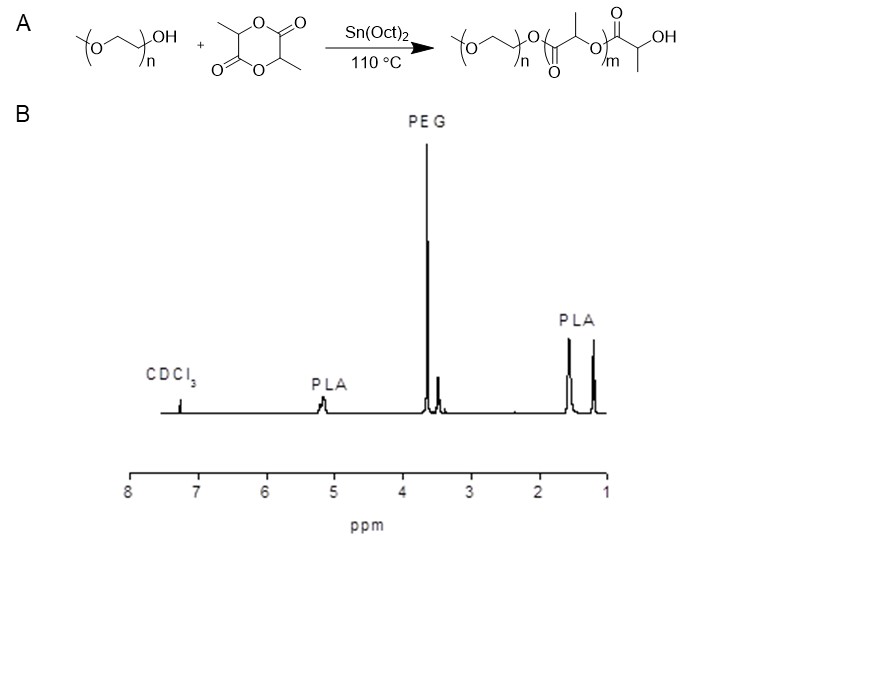


**Fig. S1.** (A) Synthetic route of PEG-PLA. (B) ^1^H NMR spectrum of PEG-PLA.


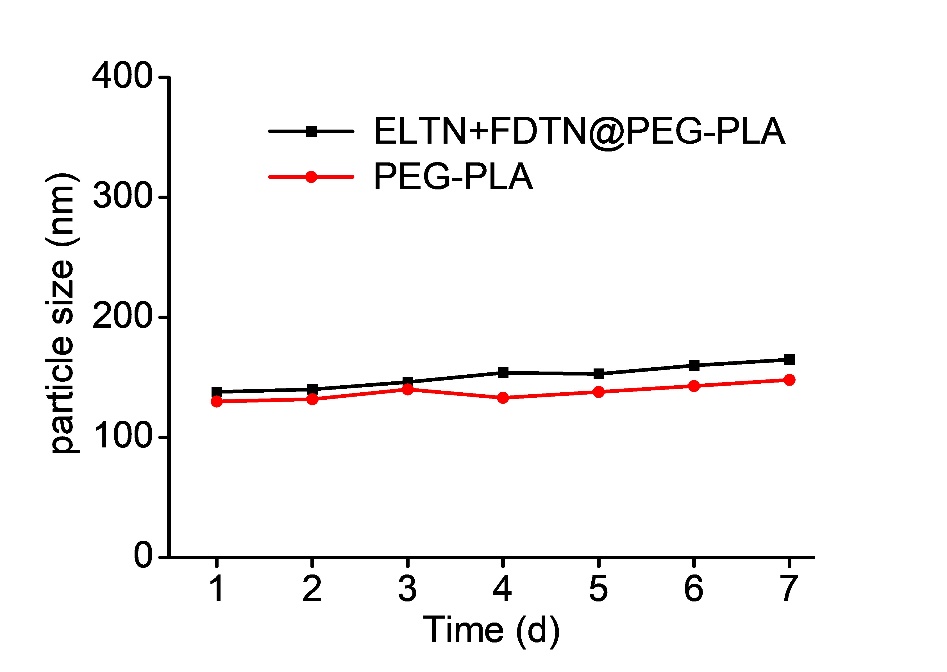


**Fig. S2.** Size alteration of NPs within 7-day incubation at RT.

**Table S1**. The IC_50_ values (μM) of ELTN and FDTN in different cell lines.

|  | H1975 | | H1650 | |
| --- | --- | --- | --- | --- |
|  | ELTN | FDTN | ELTN | FDTN |
| ELTN@PEG-PLA | >20 | --- | >20 | --- |
| FDTN@PEG-PLA | --- | 14.5 | --- | 15.2 |
| ELTN+FDTN@PEG-PLA | 4.6 | 2.3 | 5.2 | 2.6 |
| ELTN+FDTN | 3.5 | 1.7 | 2.6 | 1.3 |
| ELTN | >20 | --- | >20 | --- |
| FDTN | --- | 2.2 | --- | 1.88 |
